# Processing of performance errors predicts memory formation: Enhanced feedback‐related negativities for corrected versus repeated errors in an associative learning paradigm

**DOI:** 10.1111/ejn.14566

**Published:** 2019-10-03

**Authors:** Ellen R. A. de Bruijn, Rogier B. Mars, Rob Hester

**Affiliations:** ^1^ Department of Clinical Psychology Leiden University Leiden The Netherlands; ^2^ Leiden Institute for Brain and Cognition (LIBC) Leiden The Netherlands; ^3^ Wellcome Centre for Integrative Neuroimaging Centre for Functional MRI of the Brain (FMRIB) Nuffield Department of Clinical Neurosciences John Radcliffe Hospital University of Oxford Oxford UK; ^4^ Donders Institute for Brain, Cognition and Behaviour Radboud University Nijmegen Nijmegen The Netherlands; ^5^ Melbourne School of Psychological Sciences University of Melbourne Melbourne Vic. Australia

**Keywords:** errors, event‐related potential, feedback‐related negativity, learning, P300, performance monitoring

## Abstract

Learning from errors or negative feedback is crucial for adaptive behavior. FMRI studies have demonstrated enhanced anterior cingulate cortex activity for errors that were later corrected versus repeated errors even when a substantial delay between the error and the opportunity to correct was introduced. We aimed at identifying the electrophysiological correlates of these processes by investigating the feedback‐related negativity (FRN) and stimulus‐locked P3. Participants had to learn and recall the location of 2‐digit targets over consecutive rounds. Feedback was provided in two steps, first a color change indicated a correct or incorrect response (feedback phase) followed by presentation of the correct digit information (re‐encoding phase). Behaviorally, participants improved performance from the first to the third round. FRN amplitudes time‐locked to feedback were enhanced for corrected compared to repeated errors. The P3 in response to re‐encoding did not differ between the two error types. The finding that FRN amplitudes positively predicted memory performance is consistent with the idea that the FRN reflects prediction errors and the need for enhanced cognitive control. Interestingly, this happens early during feedback processing and not at a later time point when re‐encoding of correct information takes place. The prediction error signal reflected in the FRN is usually elicited by performance errors, but may thus also play a role in preparing/optimizing the system for memory formation. This supports the existence of a close link between action control and memory processes even when there is a substantial delay between error feedback and the opportunity to correct the error.

## Graphical abstract

Using EEG and a visual associative learning paradigm, we disentangled processing of initial negative feedback and later re‐encoding of correct information. The results showed that the amplitude of the feedback‐related negativity (FRN), but not the P3, positively predicted memory performance. This was specific for feedback processing and not at the later time point of re‐encoding. These findings support the idea that the FRN reflects prediction errors and the need for enhanced cognitive control.

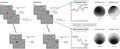

**Abbreviations**
ACC: anterior cingulate cortex
EEG: electroencephalography
EOG: electrooculogram
ERN: error‐related negativity
ERP: event‐related potential
fMRI: functional magnetic resonance imaging
FRN: feedback‐related negativity
pMFC: posterior medial frontal cortex
pre‐SMA: pre‐supplementary motor area

## 1 INTRODUCTION

Using neuroimaging methods such as electroencephalography (EEG) and functional magnetic resonance imaging (fMRI), it has been well established that areas in posterior medial frontal cortex (pMFC) including anterior cingulate cortex (ACC) and pre‐supplementary motor area (pre‐SMA) play a central role in action or performance monitoring processes, such as error detection and regulation of adaptive behavior (see e.g., de Bruijn, de Lange, von Cramon, & Ullsperger, 2009; Debener et al., 2005). Specifically, an event‐related potential (ERP) known as the error‐related negativity (ERN) is elicited immediately following errors in speeded choice reaction‐time tasks (Falkenstein, Hohnsbein, Hoormann & Blanke, 1990; Gehring, Goss, Coles, Meyer, & Donchin, 1993). When one needs to rely on external feedback to determine the correctness of a given response, for example, while learning is ongoing, a similar error‐related response can be seen at the moment of delivery of negative feedback. This stimulus‐locked error signal is known as the feedback‐related negativity (FRN; Holroyd & Coles, 2002; Miltner, Braun, & Coles, 1997; Nieuwenhuis, Holroyd, Mol, & Coles, 2004) or medial frontal negativity (MFN; Gehring & Willoughby, 2002).

The ERN is thought to result from dopamine‐based prediction errors signaling a loss of reward (Barnes, O'Connell, Nandam, Dean, & Bellgrove, 2014; de Bruijn, Hulstijn, Verkes, Ruigt, & Sabbe, 2004; de Bruijn, Sabbe, Hulstijn, Ruigt, & Verkes, 2006; Forster et al., 2017; Holroyd & Coles, 2002; Jocham & Ullsperger, 2009; Spronk et al., 2016; Zirnheld et al., 2006). Prediction errors are elicited when an expected action outcome differs from the actual outcome and function as teaching signals used to optimize motor behavior (see, e.g., Ullsperger, Danielmeier, & Jocham, 2014; Ullsperger, Fischer, Nigbur, & Endrass, 2014). Indeed, previous studies have demonstrated that the amplitude of these error‐related ERP components may be related to such fast behavioral changes such as post‐error slowing (Debener et al., 2005; Garavan, Ross, Murphy, Roche, & Stein, 2002), internalizing of stimulus–response contingencies (see, e.g., Holroyd & Coles, 2002) or implicit motor learning (van der Helden, Boksem, & Blom, 2010). Studies also indicate that the FRN scales with prediction errors with higher amplitudes for more unexpected compared to more expected outcomes (Ullsperger, Danielmeier, et al., 2014; Ullsperger, Fischer, et al., 2014).

More recently, it has been proposed that the negativities observed in the stimulus‐locked ERP component following negative outcomes are in fact a N200 elicited by unexpected events that require an increased need for cognitive control (Holroyd, Pakzad‐Vaezi, & Krigolson, 2008). As increased cognitive control is not required on expected or positive outcomes, this negative component is suppressed on these trials and therefore often referred to as the feedback correct‐related positivity or the reward positivity (Holroyd et al., 2008; Proudfit, 2015; Williams, Hassall, Trska, Holroyd, & Krigolson, 2017). This more recent interpretation of increased cognitive control fits with previous studies that have demonstrated a relationship between enhanced medial frontal negativities and subsequent behavioral adjustments (e.g., Cohen & Ranganath, 2007; Holroyd & Coles, 2002; Sallet, Camille, & Procyk, 2013; but see also Chase, Swainson, Durham, Benham, & Cools, 2011; Von Borries, Verkes, Bulten, Cools, & de Bruijn, 2013). However, it is unknown if a similar relationship between action control and learning also exists for more long‐term memory‐formation processes, that is, trials on which there is a delay between presentation of negative feedback and the next opportunity to perform correctly.

Using a visual associative learning paradigm, recent fMRI studies have shown that error‐related ACC activity predicts learning from errors, even when the time between the error and the opportunity to correct the error could vary up to 90 s (Hester, Barre, Murphy, Silk, & Mattingley, 2008; Hester, Murphy, Brown, & Skilleter, 2010). In this learning paradigm, participants have to recall the spatial locations of 2‐digit targets. The task consists of multiple rounds of responding, which enables the comparison of errors that are repeated in the next round to errors that are corrected—and thus digits that are remembered correctly—in the following round. Compared to repeated errors, corrected errors elicited increased activation in ACC, which was associated with enhanced activity in hippocampus (Hester et al., 2008, 2010).

To date, however, the ERP components specific for processing negative feedback (FRN) and updating of context or working memory representations (the stimulus‐locked P3; see e.g., Donchin & Coles, 1988; Johnson, 1986; Polich, 2007) have not been investigated using this learning paradigm. Importantly, the excellent temporal resolution of EEG methodology may provide more insight into the underlying processes and how they develop over time. The aim of the current study therefore was to disentangle processing of negative feedback about one's actions and encoding of the correct information during associative learning by means of measuring ERPs. We focused on the FRN elicited by negative (i.e., error) feedback and the P3 elicited by the presentation of correct target information (i.e., re‐encoding). A short delay between feedback onset and encoding allowed us to dissociate between the ERPs generated by the two events. Based on the previous fMRI work using this paradigm and the presumed role of the FRN in prediction error signaling and learning, we expected increased FRN amplitudes for corrected compared to repeated errors following feedback. For the P3, we also expected enhanced amplitudes for corrected errors in response to the re‐encoding information, because of the component's role in context updating and working memory.

## 2 MATERIAL AND METHODS

### 2.1 Participants

Eighteen healthy volunteers (11 females; mean age = 24.4 years; range = 21–29 years) participated in the experiment for course credits. All participants provided written informed consent. Procedures were in accordance with the Declaration of Helsinki and approved by the local Ethics Committee of the Radboud University in Nijmegen, the Netherlands.

### 2.2 Design

**Figure 1:**
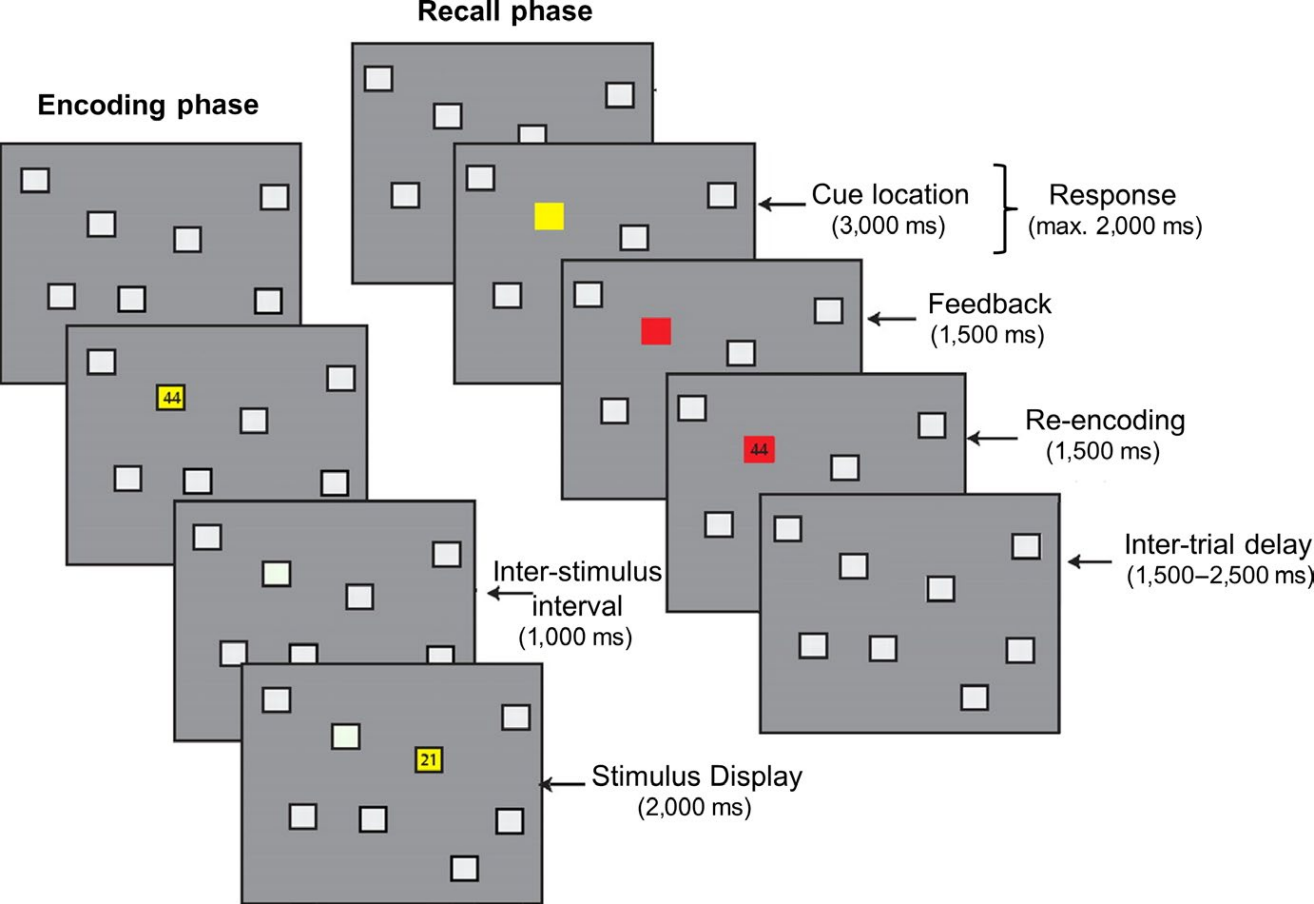
Trial timing of the EEG version of the learning from error task with trial sequences shown for both the encoding (left) and the recall phase (right) [Colour figure can be viewed at http://wileyonlinelibrary.com]

Participants performed the learning from errors task, an associative learning task in which they had to recall the spatial locations of 2‐digit targets (see Figure 1). The task (a modified version of the one used in Hester et al., 2008) began with an encoding phase in which eight locations designated as gray squares were presented simultaneously on a black background. The locations of the squares on the background were selected in a quasi‐random fashion from an 8 × 8 matrix, with two locations randomly chosen from each of the four quadrants of the display. At the commencement of the encoding phase, each location in turn had superimposed upon it a 2‐digit number (c.f., Hester et al., 2008). The number remained visible for 2 s and was followed by an inter‐stimulus interval of 1 s. The digits of each number consisted of 1, 2, 3 or 4, and participants identified the number by entering each digit using the appropriate buttons on a response box. Two‐digit numbers were used to reduce the probability of guessing the correct answer to 6% (Hester et al., 2008). The encoding phase lasted 24 s in total and was followed by a 1,500‐ms interval prior to the start of the recall phase. Following the encoding phase in which numbers were shown for each of the eight locations, a series of recall trials were presented. During a recall trial, one of the eight locations was highlighted in yellow, cueing the participant to respond with the 2‐digit number associated with that location. Participants were required to respond within 2 s, after which feedback was presented for 1,500 ms. Feedback provided only the validity of the response: the location square turned blue to indicate a correct response or red to indicate an incorrect response.

Unlike the original associative learning task from Hester et al. (2008), presentation of the correct number followed feedback presentation, thus introducing a delay of 1,500 ms between the two events. The correct number was shown upon the colored (red or blue) background. This delay allowed us to dissociate between the ERP components associated with negative feedback processing (FRN) and encoding of the correct information (P300). The correct number remained on the screen for 1,500 ms. This was followed by a random inter‐trial delay between 1,500 and 2,500 ms after which the next recall trial started. All eight location squares remained on screen during each epoch of the recall trials. Each location in a block of eight trials was highlighted once for recall, before being highlighted a second and third time, all in different pseudorandom orders (Hester et al., 2008). This created three rounds of eight recall trials within each block. Six blocks of the encoding/recall cycle were administered to each participant, with each block involving a different array of locations and 2‐digit numbers. No location in the array was used more than once throughout the six runs, and the 2‐digit numbers were not repeated on consecutive blocks.

### 2.3 Different error types

The main aim of the current study was to compare corrected errors to repeated errors. Corrected errors were defined as trials that were responded to incorrectly in round *N*, but were correctly responded to in round *N* + 1. Repeated errors were those trials that were responded to incorrectly in both round *N* and the next round (*N* + 1). Both these error types were compared to consecutive correct recall trials, that is, trials that were responded to correctly in both round N and *N* + 1. Please note that all ERP signals are time‐locked to the events in block *N*, but are thus categorized on the basis of future performance (i.e., in block *N* + 1).

### 2.4 Electrophysiological recording and data preprocessing

The electroencephalogram (EEG) was recorded from 27 tin electrodes mounted in an elastic electrode cap (Electrocap International). Electrodes were placed at seven midline (FPz, AFz, Fz, FCz, Cz, Pz and Oz) and twenty lateral locations (FP1/2, F3/4, F7/8, FC5/6, T3/T4, T5/6, C3/4, CP5/6, P3/4 and O1/2) in accordance with an extended version of the international 10–20 system. All signals were referenced to the left mastoid but were later offline re‐referenced to the average of both mastoids. The vertical electrooculogram (EOG) was recorded bipolarly from electrodes placed above and below the right eye. The horizontal EOG was recorded bipolarly from electrodes lateral to both eyes. All electrode impedances were kept below 5 kΩ‎. The EEG and EOG signals were amplified using a time constant of 8 s (high pass .02 Hz) and were filtered offline low pass at 15 Hz. All signals were digitized with a sampling rate of 500 Hz.

Electroencephalogram data were further analyzed offline using Brain Vision Analyzer 2.0 (Brain Products). Eye movements were corrected using the Gratton, Coles and Donchin method (Gratton, Coles, & Donchin, 1983) followed by artifact rejection. Stimulus‐locked ERPs were baseline‐corrected relative to a 200 ms pre‐stimulus baseline for both onset of feedback and encoding. For corrected errors, repeated errors and repeated correct recalls, epochs were averaged separately and time‐locked to feedback and encoding onset, starting 200 ms before and ending 700 ms after stimulus onset.

### 2.5 Analyses

First, trials with incomplete (3.1%) or too late (2.5%) responses, as well as trials on which a participant reversed the correct numbers (5.0%), were removed from all analyses, as it is very uncertain in these instances if the error was resulting from actual incomplete knowledge. Behavioral analyses focused on the percentage of correct responses in the three rounds, the percentage of corrected errors from round 1–2 and from round 2–3, as well as the number of repeated correct recalls from round 1–2 and from round 2–3. Individual mean percentages were entered into repeated‐measures general linear models with Round (three levels: 1, 2 and 3) and Change (two levels: round 1–2 and round 2–3) as possible within‐subject factors.

To investigate the FRN and the P3 for the two error types, difference waves were calculated between Repeated Errors and consecutive Correct Recalls (i.e., correct recalls that were also correctly recalled in the next round) and between Corrected Errors and consecutive Correct Recalls. This was done for both individual averages relative to the onset of the feedback and to the onset of encoding. FRN amplitude was determined on the difference waves for feedback onset as the most negative peak in the 200‐ to 400‐ms time window following feedback and re‐encoding presentation at electrodes Fz, FCz, Cz and Pz. P3 amplitudes were determined on the difference waves relative to re‐encoding and feedback onset as the most positive peak in the 300‐ to 700‐ms time window after presentation of the correct number at the same four midline electrodes.

In addition to the difference wave analyses, FRN and P3 analyses were conducted on the three separate correctness conditions (i.e., consecutive Correct Recall, Corrected Errors, and Repeated Errors). As clear FRN peaks were not always evident on Correct Recall trials, FRN amplitudes were determined as mean amplitude in the 250‐ to 300‐ms time interval following feedback onset for these analyses (Walentowska, Moors, Paul, & Pourtois, 2016). P3 amplitude was again determined as the most positive peak in the 300‐ to 700‐ms time window after re‐encoding onset. Individual mean amplitudes were entered into repeated‐measures general linear models with Error Type (two levels: corrected errors, repeated errors or three levels with the consecutive correct recall condition included) and Electrode (four levels: Fz, FCz, Cz and Pz) as within‐subject factors.

Finally, to investigate more general attention‐related processes, analyses were conducted for the feedback‐locked N1 and P2 component. N1 amplitude was defined as the most negative peak in the 0‐ to 150‐ms time window following feedback onset at electrodes Fz and Pz where amplitudes were maximal. The N2 was defined as the most positive peak in the 150‐ to 250‐ms time window following feedback onset at electrode Cz where amplitudes were maximal. Individual mean amplitudes were entered into repeated‐measures general linear models with Error Type (three levels: consecutive Correct Recall, Corrected Errors and Repeated Errors) as within‐subject factor.

## 3 RESULTS

### 3.1 Behavioral results

**Figure 2:**
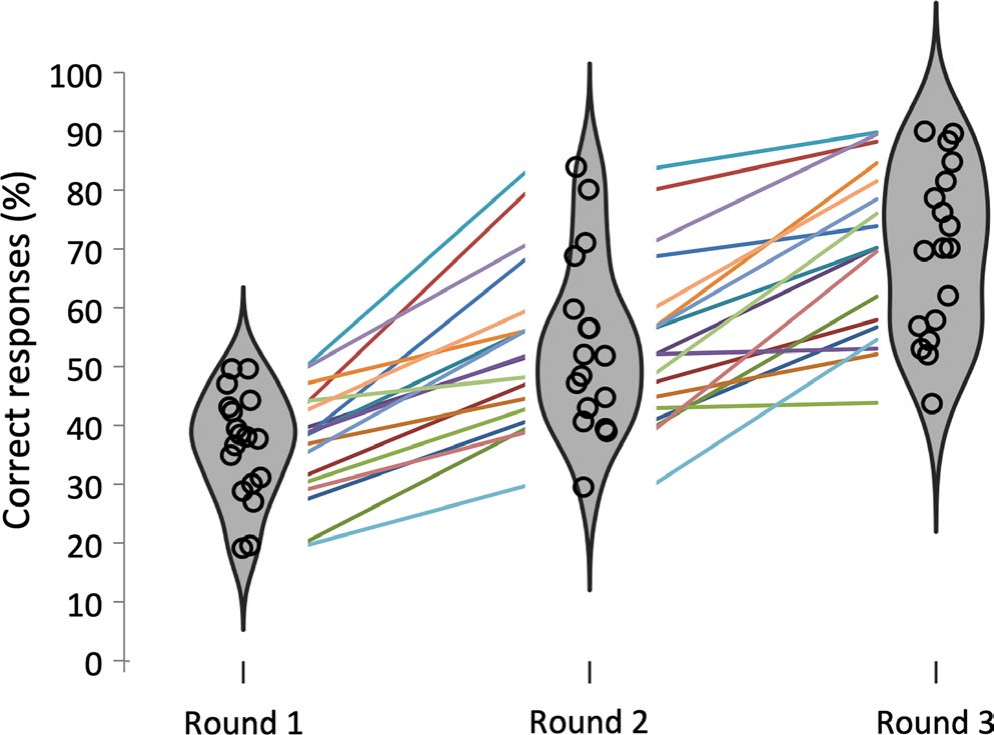
Violin plots showing the distribution for percentage of correct responses for the three different rounds. Circles represent individual means, and colored lines represent individual changes in performance [Colour figure can be viewed at http://wileyonlinelibrary.com]

Recall performance improved from the first to the third round (see also Figure 2) as reflected in a main effect of Round for percentage of correct responses, *F*_2,16_ = 94.95, *p *<* *.001, $\eta_{p}^{2}$ = .87. Participants responded correctly on more trials in round 2 (54.2%) compared to round 1 (37.1%), *F*_1,17_ = 65.69, *p *<* *.001, $\eta_{p}^{2}$ = .79, and the highest percentage of correct responses was observed in round 3 (69.7%), *F*_1,17_ = 144.29, *p *<* *.001, $\eta_{p}^{2}$ = .90.

**Figure 3:**
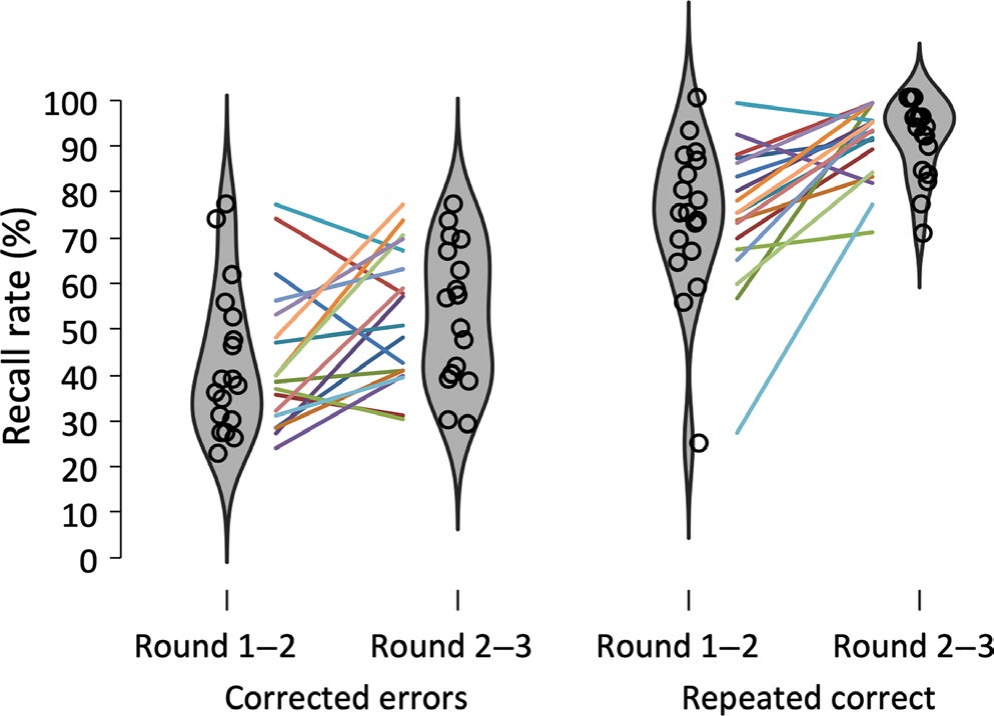
Violin plots showing the distribution for mean recall error correction rates (expressed as a percentage of total errors in round *N* that were corrected in round *N* + 1) and mean repeated correct recall rates (expressed as a percentage of total correct recalls in round *N* that were again recalled correctly in round *N* + 1). Circles represent individual means, and colored lines represent individual changes in performance [Colour figure can be viewed at http://wileyonlinelibrary.com]

Figure 3 depicts the violin plots for percentage of corrected errors (expressed as a percentage of total errors in round *N* that were corrected in round *N* + 1) and repeated correct recall rates (expressed as a percentage of total correct recalls in round *N* that were also recalled correctly in round *N* + 1) from the first to the second and from the second to the third round. The percentage of corrected trials improved over the rounds (from 42.4% to 52.6%) as reflected in a main effect of Change, *F*_1,17_ = 6.35, *p *=* *.022, $\eta_{p}^{2}$ = .27. The same pattern was found for repeated correct recalls (from 74.0% to 91.1%), *F*_1,17_ = 22.40, *p *<* *.001, $\eta_{p}^{2}$ = .57.

### 3.2 ERP results: Feedback onset

**Figure 4:**
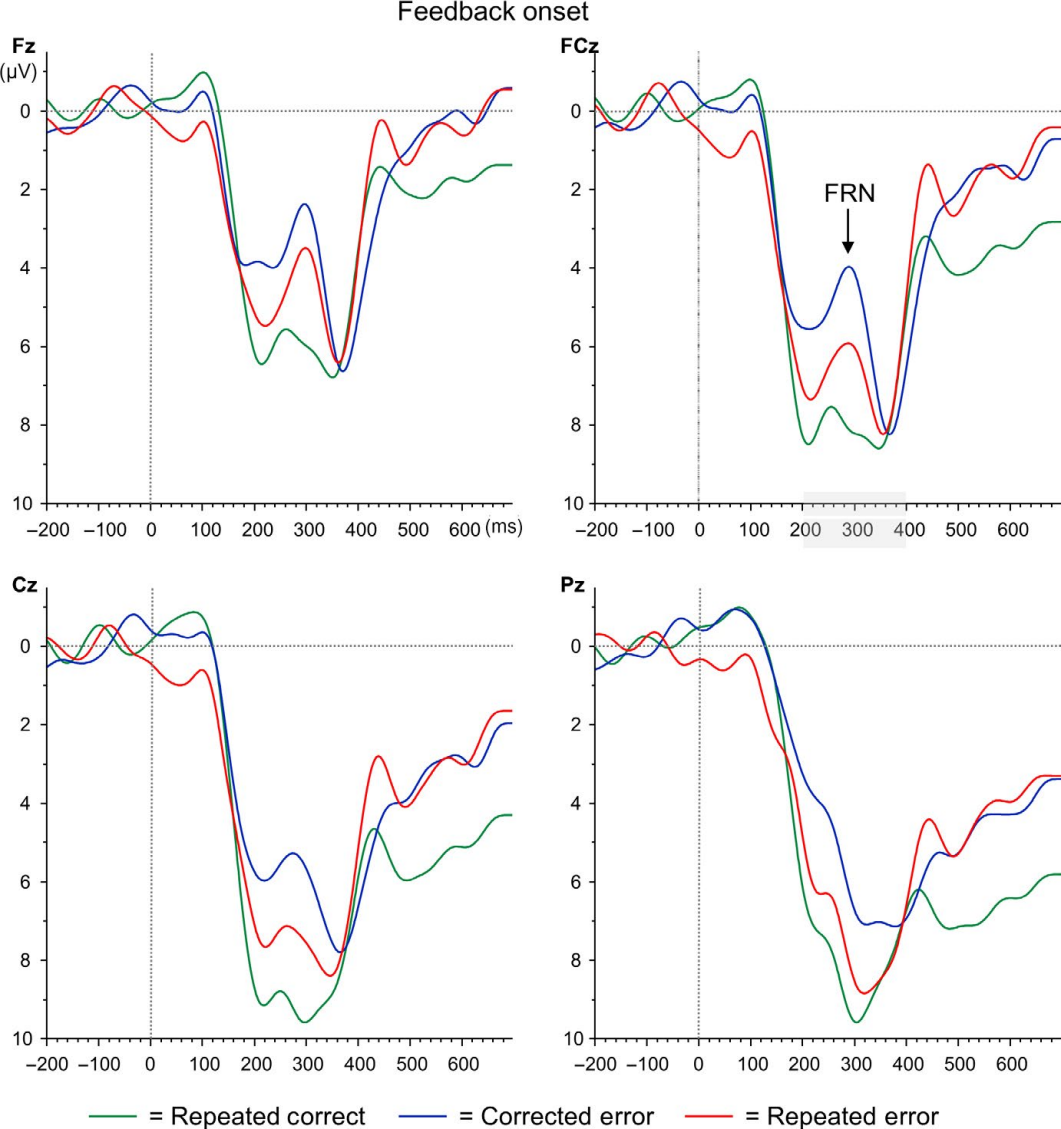
Grand average ERP waveforms time‐locked to feedback onset (= 0 ms) for electrodes Fz, FCz, Cz and Pz. Repeated correct trials are depicted in green, corrected errors in blue and repeated errors in red [Colour figure can be viewed at http://wileyonlinelibrary.com]

The grand average waveforms time‐locked to feedback onset are presented in Figures 4 and 6a and show a reward positivity on positive/correct feedback, but increased negativities with a frontocentral distribution (see Figure 6b) around 300 ms following stimulus onset for negative/error feedback. The FRN analyses time‐locked to feedback onset revealed a main effect for Error Type, *F*_1,17_ = 6.39, *p *=* *.022, $\eta_{p}^{2}$ = .27, with increased FRN amplitudes for corrected (−7.25 μV) compared to repeated errors (−5.44 μV). Although FRN amplitudes were numerically largest at FCz (−6.87 μV), the main effect for Electrode did not reach significance, *F*_3,15_ = 2.56, *p *=* *.094). The interaction between Error Type and Electrode was not significant either (*F <* 1).

The P3 analyses time‐locked to feedback onset did not reveal any significant main effects (main effect Electrode: *p *=* *.136; main effect Error Type: *p *=* *.745). The interaction between the two was not significant either (*F* < 1).

The additional analyses aimed at comparing FRN amplitudes for errors to correct trials, demonstrated a main effect of Electrode, *F*_3,15_ = 16.60, *p *<* *.001, $\eta_{p}^{2}$ = .77 and a main effect of Error Type, *F*_2,16_ = 6.89, *p *=* *.007, $\eta_{p}^{2}$ = .46. The interaction between the two was not significant, *F*_6,12_ = 2.85, *p *=* *.058, $\eta_{p}^{2}$ = .59. Pairwise comparisons demonstrated largest, that is, most negative, FRN amplitudes at Fz (4.29 μV) compared to FCz (6.25 μV), Cz (7.54 μV), and Pz (7.49 μV; all *p*s* *<* *.001). Follow‐up tests of the main effect of Error Type showed that FRN amplitudes were most negative for Corrected Errors (4.51 μV) compared to consecutive Correct Recall trials (8.31 μV; *p *=* *.003) and Repeated Errors (6.36 μV; *p *=* *.048). The latter two did not differ significantly from one another (*p *=* *.149).

The analyses for the feedback‐locked N1 showed no effects of Error Type (both Fs < 1). The P2 analyses demonstrated a main effect of Error Type, *F*_2,16_ = 4.37, *p *=* *.031, $\eta_{p}^{2}$ = .35. Follow‐up Helmert contrasts showed that P2 amplitudes were most positive for consecutive Correct Recall trials (10.32 μV; *p *=* *.029). Importantly, Corrected Errors (7.25 μV) and Repeated Errors (8.51 μV) did not differ significantly from each other (*p *=* *.113).

### 3.3 ERP results: Re‐encoding onset

**Figure 5:**
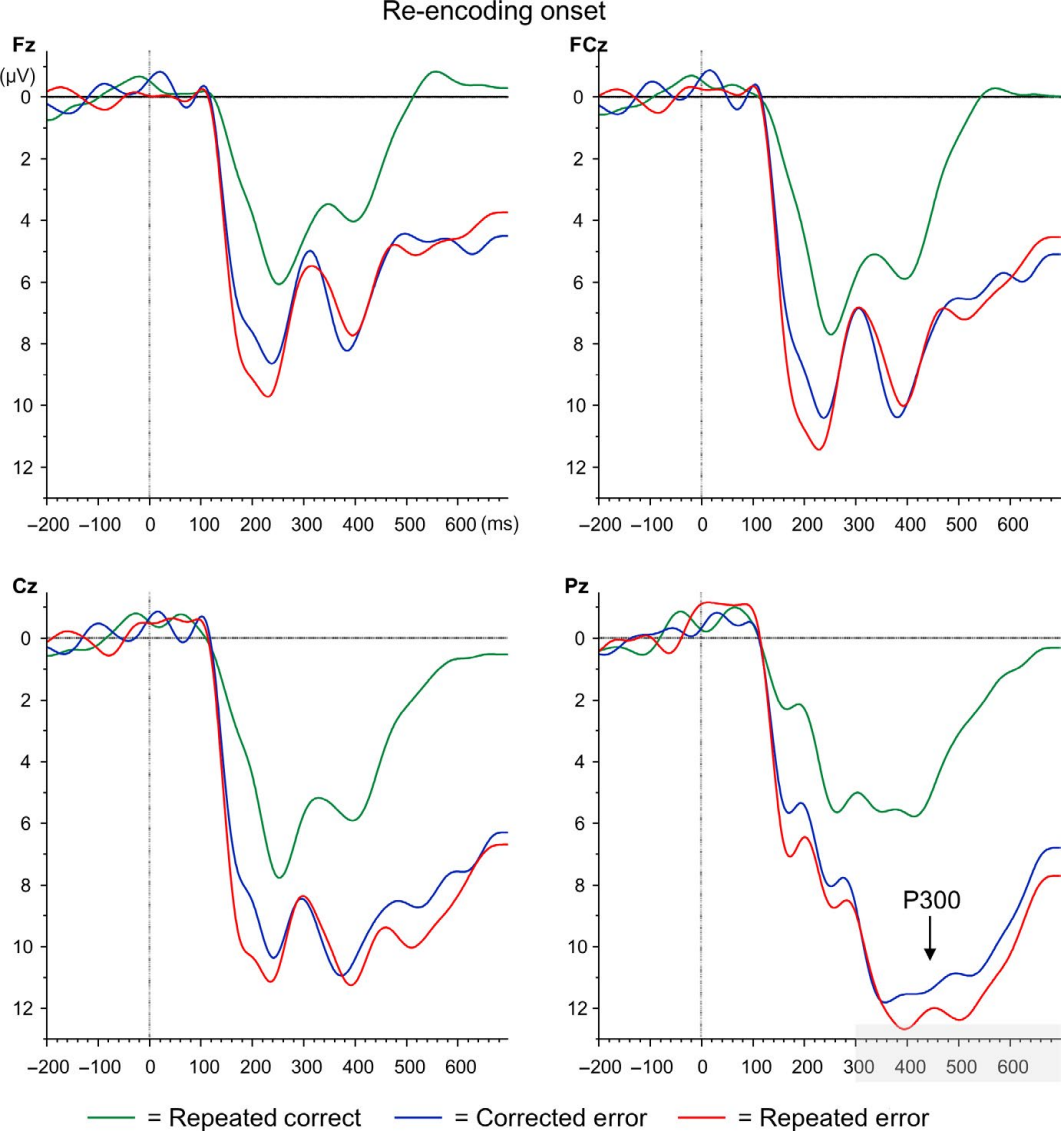
Grand average ERP waveforms time‐locked to re‐encoding onset (= 0 ms) for electrodes Fz, FCz, Cz and Pz. Repeated correct trials are depicted in green, corrected errors in blue and repeated errors in red [Colour figure can be viewed at http://wileyonlinelibrary.com]

**Figure 6:**
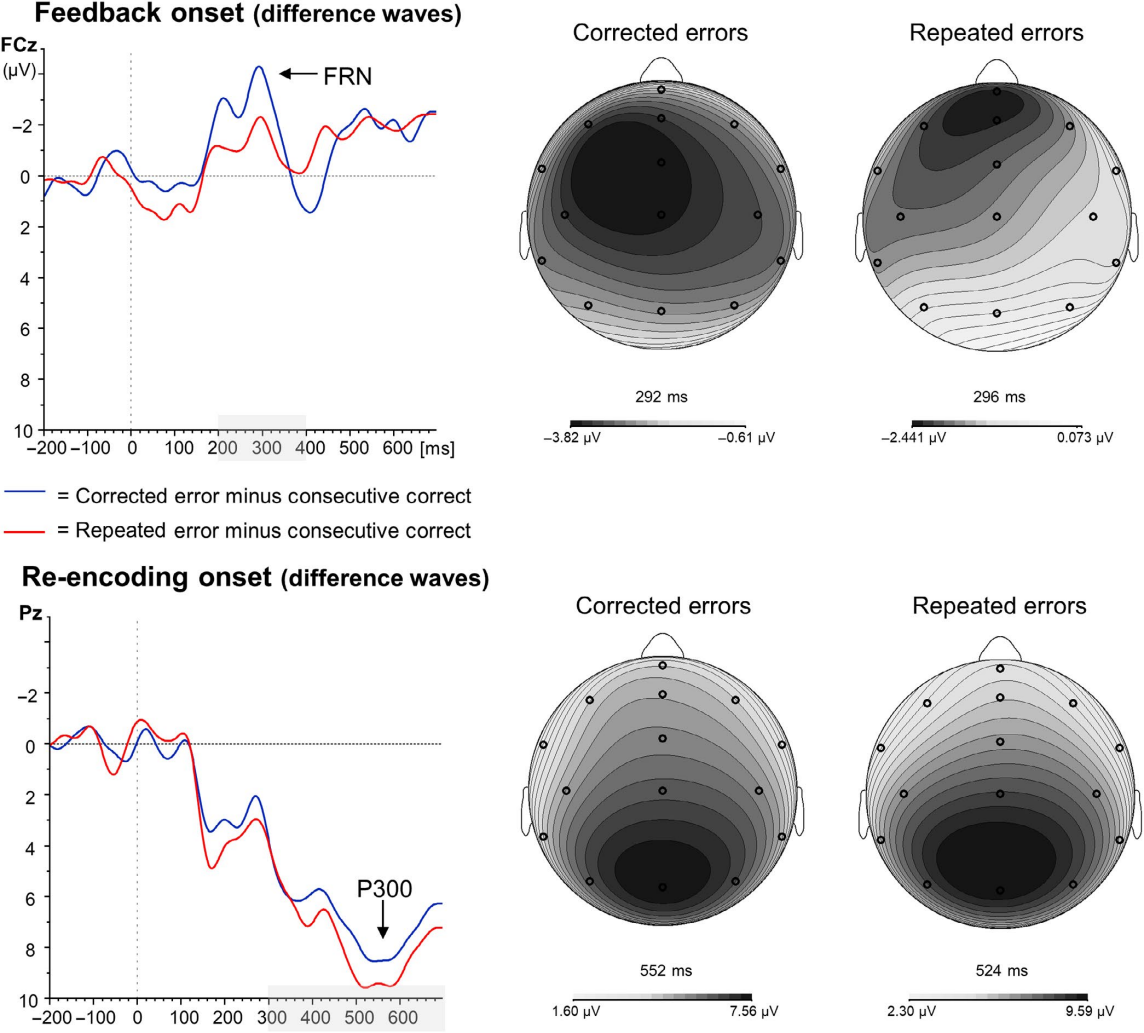
Left panel: Difference waveforms for the two error types relative to the consecutive correct condition for feedback (top) and re‐encoding onset (bottom). Right panel: Topographical distributions of the FRN (top) and P300 (bottom) at maximal peak onset of the difference wave [Colour figure can be viewed at http://wileyonlinelibrary.com]

The P3 analyses time‐locked to re‐encoding onset (see Figures 5 and 6a) only revealed a main effect of Electrode, *F*_3,15_ = 8.57, *p *=* *.001, $\eta_{p}^{2}$ = .63. Follow‐up contrasts showed that P3 amplitude was maximal at Pz (14.15 μV) compared to the other electrodes (all *p*s* *<* *.032), which is in line with the posterior distribution of the component as depicted in Figure 6b. Neither the main effect of Error Type (*F* < 1) nor the interaction between Error Type and Electrode reached significance, *F*_3,15_ = 1.81, *p *=* *.189.

Similar as the P3 analyses, the FRN analyses time‐locked to re‐encoding onset only revealed a main effect of Electrode, *F*_3,15_ = 9.07, *p *=* *.001, $\eta_{p}^{2}$ = .65. Follow‐up analyses showed that FRN amplitudes did not differ between Fz (−0.18 μV) and FCz (−0.22 μV; *p *=* *.896), but FRN amplitude at FCz was significantly more negative than Cz (0.81 μV; *p *=* *.002) and Pz (1.67 μV; *p *<* *.001). FRN amplitude was smallest at Pz (all *p*s* *<* *.034). Neither the main effect of Error Type (*F* < 1) nor the interaction between Error Type and Electrode reached significance, *F*_3,15_ = 2.97, *p *=* *.065.

The additional analyses aimed at comparing P3 amplitudes for errors to correct trials demonstrated a main effect of Electrode, *F*_3,15_ = 25.72, *p *<* *.001, $\eta_{p}^{2}$ = .84 and a main effect of Error Type, *F*_2,16_ = 11.66, *p *=* *.001, $\eta_{p}^{2}$ = .59. Also, the interaction between the two was significant, *F*_6,12_ = 18.29, *p *<* *.001, $\eta_{p}^{2}$ = .90. As expected, pairwise comparisons demonstrated that P3 amplitudes were maximal at electrode Pz (13.52 μV) compared to Fz (8.86 μV, *p *<* *.001), FCz (11.06 μV, *p *<* *.001) and Cz (12.46 μV; *p *=* *.013). At electrode Pz, P3 amplitudes for consecutive Correct Recall trials (7.96 μV) were significantly smaller than Corrected Errors (15.85 μV, *p *<* *.001) and Repeated Errors (16.79 μV, *p *<* *.001). Corrected and Repeated Errors did not differ significantly (*p *=* *.204).

## 4 DISCUSSION

The aim of the present study was to investigate the electrophysiological correlates of learning from errors, by comparing FRN and P300 amplitudes for corrected and repeated errors at different stages of information processing. As expected from previous fMRI studies using the same paradigm, participants showed improved performance from round 1 to round 3, reflecting the ability to learn from errors in this task (c.f., Hester et al., 2008, 2010). FRN amplitudes relative to feedback onset were larger for corrected than for repeated errors, but P300 amplitudes relative to re‐encoding onset did not differ between the two error types.

The increased FRN amplitudes following incorrect feedback for errors that were subsequently corrected compared to errors that were repeated are consistent with previous fMRI studies demonstrating increased ACC involvement for corrected errors (Hester et al., 2008, 2010). The FRN is thought to originate from areas in posterior medial frontal cortex (pMFC), specifically ACC and pre‐supplemental motor area (see, e.g., de Bruijn et al., 2009; Holroyd et al., 2004). The FRN has therefore originally been interpreted as a reflection of processing of erroneous or negative feedback thought to play a central role in reinforcement learning (Holroyd & Coles, 2002). The finding of enhanced FRNs for corrected errors shows that FRN amplitude in the current study was predictive of learning even when a substantial delay between onset of negative feedback and the next opportunity to correct the error was introduced.

Interestingly, this relationship with performance was not observed for the P3 amplitude generally assumed to play a central role in the updating of context or working memory presentations following unexpected events (see e.g., Donchin, 1981; Donchin & Coles, 1988). One explanation for this is the well‐known finding that P3 amplitudes decrease with increasing task difficulty or memory load (for a review see Kok, 2001). Kok (2001) proposed that—in high demand situations—the P3 may be less sensitive than actual performance measures and might saturate earlier. The currently observed levels of performance do suggest that the associative learning paradigm places high demands on the participants. Also, compared to repeated correct trials, the P3 is enhanced for both corrected and repeated errors during the re‐encoding phase. This indicates that participants indeed interpret these events as more salient and task‐relevant (see, e.g., Donchin, 1981; Duncan‐Johnson & Donchin, 1977), but combined with the greater task difficulty and high memory load the currently observed similar P3 amplitudes for both error types may represent a ceiling effect.

The FRN and P3 findings combined pose an intriguing finding as it shows that processing of the negative feedback signal is predictive for future learning and not processing at a later stage when the relevant information can be re‐encoded and updated. Note that the analyses on the feedback‐locked N1 and P2 components did not provide support for more general attention‐related processes that might alternatively explain the currently found difference between the two error types. These findings fit well with the recent interpretation that the FRN may be a stimulus‐locked N200, which is increased when more cognitive control is required (Holroyd et al., 2008; Mulligan & Hajcak, 2018). Trials on which more cognitive control is issued thus result in enhanced memory performance later on even when this is accompanied by a lack of apparent differences between processing of corrected and repeated errors during the re‐encoding phase. Also, the current pattern of results is consistent with the idea that the FRN carries a prediction error signal elicited by unexpected events, which is used for optimizing future performance (see, e.g., Ullsperger, Danielmeier, et al., 2014; Ullsperger, Fischer, et al., 2014). Together these results show that the FRN elicited by an early binary feedback signal is predictive of future memory performance even in the absence of the necessary information needed to update the context such that relevant information can be re‐encoded and memorized.

One limitation of the current study is that we do not know if there was a specific variable that determined if an error would be corrected or not. For example, a participant might have been unsure about two possible answer options for one trial, while completely uncertain (and thus a higher number of possible answer options) for another trial. A participants’ response during a corrected error may have therefore resulted from one of two possible answer options being eliminated by the presentation of the incorrect feedback signal. The signal not only told them that their chosen answer was wrong, but also immediately informed them about the alternative and thus correct option. For repeated errors, uncertainty may have been higher and participants perhaps did not have a concrete correct answer available. In that case, the negative feedback signal is more expected and may thus result in reduced FRN amplitudes compared to corrected errors as it is known that FRN amplitudes scale with expectancy (Ullsperger, Danielmeier, et al., 2014; Ullsperger, Fischer, et al., 2014). However, it is important to note that the P3 is also known to scale with expectancy, subjective probability or surprise with increased P3 amplitudes for events that are more surprising (e.g., Donchin & Coles, 1988; Mars et al., 2008; Nieuwenhuis, Aston‐Jones, & Cohen, 2005). If corrected and repeated errors would thus have differed with regard to expectancy or surprise, smaller P3 amplitudes would have been predicted following feedback for repeated errors. Our analyses showed that this is not the case. Although we can thus not completely rule out a possible effect of subjective expectancy, our results seem to fit particularly well with previous studies that provided evidence for the role of the FRN in enhanced recruitment of cognitive control following unexpected events (see, e.g., Holroyd et al., 2008).

To conclude, the frontocentrally distributed negativity that is elicited in the current associative learning task after negative/error feedback is likely to reflect recruitment of cognitive control that enhances performance even after a substantial delay in time. Long‐term learning may thus also rely on processes generally involved in performance monitoring, prediction error signaling and relatively fast behavioral adjustments.

## CONFLICT OF INTEREST

None of the authors reported competing financial interests or potential conflicts of interest.

## AUTHOR CONTRIBUTIONS

EDB, RBM and RH designed the study. EDB and RBM collected and analyzed the data. EDB drafted the paper. EDB, RBM and RH revised and finalized the manuscript.

## DATA AVAILABILITY STATEMENT

According to the Guidelines for the archiving of academic research for faculties of behavioral and social sciences in the Netherlands, researchers in the institute are obliged to archive their published research data for a minimum of 10 years in DataverseNL. Within 1 month after the definitive publication of a manuscript, a researcher provides a “publication package,” compiled according to this instruction.
